# The Use of Lattice Radiation Therapy (LRT) in the Treatment of Bulky Tumors: A Case Report of a Large Metastatic Mixed Mullerian Ovarian Tumor

**DOI:** 10.7759/cureus.389

**Published:** 2015-11-24

**Authors:** Jesus Manuel Blanco Suarez, Beatriz E Amendola, Naipy Perez, Marco Amendola, Xiaodong Wu

**Affiliations:** 1 Radiation Oncology, Hospital Universitario de Gran Canaria Doctor Negrín; 2 Miami Neuroscience Center, Innovative Cancer Institute; 3 Innovative Cancer Institute

**Keywords:** lattice radiotherapy (lrt), spatially fractionated radiation therapy (grid), mullerian ovarian tumor, metastasis, carcinosarcoma, bulky tumor, volumetric modulated arc therapy (vmat), igrt, ovarian metastatic mass, ebrt

## Abstract

The objective of this teaching case is to report the excellent results of using lattice radiation therapy (LTR) for the treatment of a large metastasis from ovarian carcinosarcoma. This new technical concept extrapolates the traditional spatially fractionated radiation therapy (GRID) technique to advanced three-dimensional (3D) high-dose radiation therapy using modern instrumentation in radiation oncology. We report a case of a 61-year-old female with a large metastatic mass from ovarian carcinosarcoma treated by this procedure with excellent clinical and image-based follow-up results for more than four years.

## Introduction

Carcinosarcomas (also known as malignant mixed Müllerian* *tumors) are rare and highly aggressive epithelial malignancies that contain both malignant sarcomatous and carcinomatous elements. Up to 90% of ovarian carcinosarcomas will have disease that has spread beyond the ovary, usually involving the peritoneum. Ovarian carcinosarcomas typically occur in postmenopausal women at a median age of 65 years [1].

Risk factors for ovarian carcinosarcoma are obesity, nulliparity, exogenous estrogen use, tamoxifen therapy, and prior pelvic irradiation (5-30% of the patients) [2].

Symptoms of vaginal bleeding, pelvic mass, and lower abdominal pain are typically found in gynecological tumors. The tumor in the ovary is often diagnosed at the time of peritoneal spread and presents as a pelvic mass with peritoneal carcinomatosis.

The prognosis for ovarian carcinosarcoma is worse than for uterine carcinosarcoma and high-grade ovarian carcinomas of a similar FIGO stage [3]. Most of them present as advanced disease and the median overall survival ranges from 7 to 27 months. The strongest prognostic factor is determined by FIGO staging [4]. Some reports indicate that complete cytoreduction, advanced age, grade of the sarcomatous component, and the use of adjuvant chemotherapy are the most important prognostic factors [4-5].

Until recently, gynecological carcinosarcomas were considered as a subtype of sarcoma and treated as such. However, carcinosarcomas are now known to be metaplastic carcinomas; therefore, they should be treated as endometrial or ovarian high-risk carcinomas, despite the lack of specific data. Currently, there is no clear evidence to establish a consensus concerning guidelines for therapeutic management of carcinosarcomas with radiation therapy.

For ovarian carcinosarcomas and for other ovarian epithelial cancers, the mainstay of treatment is surgical cytoreduction with a total abdominal hysterectomy, bilateral salpingo-oophorectomy, omentectomy, aspiration of abdominal fluid, pelvic and para-aortic lymph node dissection, and tumor debulking followed, even in early stage, by chemotherapy. Retrospective studies have reported an improved outcome for patients undergoing an optimal debulking surgery without residual disease (less than 1.5 cm).

The use of radiotherapy without surgery has not been demonstrated to increase survival, although this is an indication for palliation and even to reduce the number of local recurrences [6]. Adjuvant external beam irradiation (EBRT) has not shown any overall survival benefit but has been reported to decrease local recurrences and to increase disease-free survival [5]. In patients with an early stage tumor, the role of radiotherapy remains unknown.

There is a novel technique in the field of EBRT that extrapolates the traditional 2D GRID procedure to advanced three-dimensional (3D) high-dose radiation therapy: lattice radiation therapy (LTR). It is thought that LRT would potentially improve the response of bulky tumors by triggering the bystander effect in the neighbor cells [7]. With LRT, a very high-dose of radiation is concentrated in small spheres called vertices. They are located inside the tumor in a way that a convenient dosimetric distribution allows keeping a conventional dose in the periphery of the tumor, thus not affecting the tolerance dose of the organs at risk.

We have had experience with this technique in more than 20 patients with excellent response in diverse areas of the body, including the thorax, abdomen, pelvis, head and neck, and extremities, for a different diagnosis. We present here a case report of a patient with a large abdominal mass treated with LRT diagnosed four years ago with follow-up results over more than three years after treatment.

## Case presentation

Informed patient consent was obtained for treatment. No identifying patient information is disclosed in this study.

A 61-year-old female presented with abdominal pain and increased abdominal girth. She underwent a CT scan of the abdomen and pelvis in August 2011, which revealed a large complex mass extending from the pelvis, likely from the left adnexa. She then underwent exploratory laparotomy, a total abdominal hysterectomy, bilateral salpingo-oophorectomy, and omentectomy (TAH/BSO). The surgical pathology report demonstrated a poorly differentiated malignant Müllerian tumor, including serous and clear cell types involving bilateral ovaries, fallopian tubes, uterine serosa, and omentum. Pelvic masses measured 7 cm, 6.5 cm, and 28 cm in the greatest dimensions, respectively. Peritoneal fluid washings were positive for malignant cells and rare cells consistent with an adenocarcinomatous component of the malignant mixed Müllerian tumor (Stage III-C). She received adjuvant chemotherapy until January 2012 with complete response. Follow up PET-CT in February 2012 showed no evidence of residual or recurrent malignancy.

**Figure 1:**
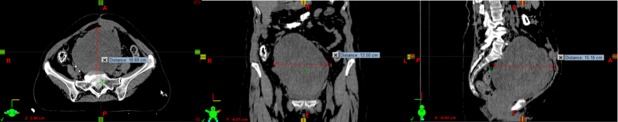
Diagnostic CT Scan of the abdomen-pelvis CT scan of the abdomen and pelvis showing a large heterogeneous pelvic mass with more than 14 cm diameter and a volume of 1495 cc as well as the tumor implants along the right psoas muscle. (November 2013)

The patient received multiple regimens of adjuvant chemotherapy, including bevacizumab, doxorubicin, gemcitabine, and Abraxane, over several months. In November 2013, two years after surgery, she was reevaluated because of a rapidly growing pelvic mass despite chemotherapy. She complained of right pelvic pain with intermittent radiation to the right leg, VAS (visual analog scale) pain level was 4/10. CT scan of the abdomen and pelvis at that time showed an interval increase in the size of the very large heterogeneous pelvic mass with more than a 14 cm diameter and a volume of 1,495 cc as well as tumor implants along the right psoas muscle and adjacent to the gallbladder as shown in Figure 1.

**Figure 2:**
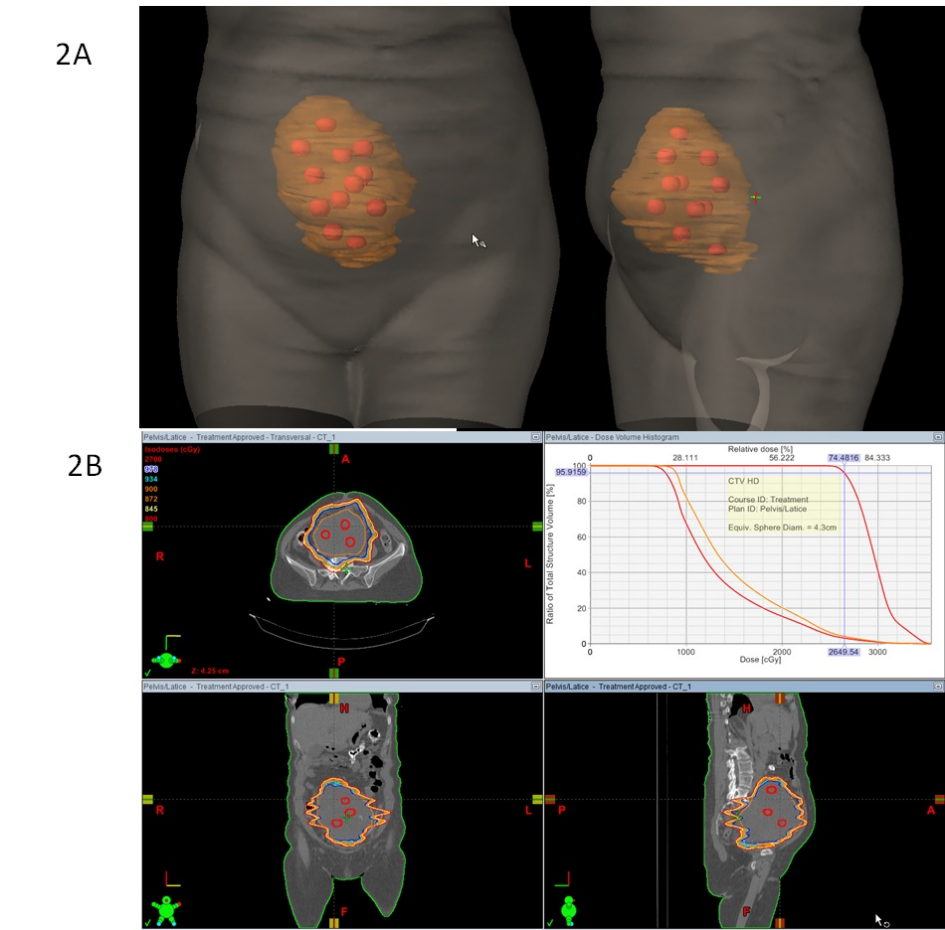
Volumetric treatment images 2A) Anterior and lateral views of  volumetric images showing total volume of the lesion receiving 3 Gy/fraction (orange) and vertices receiving 9 Gy/fraction (red points). 2B)  Lattice radiation therapy (LTR) treatment planning: vertices receiving 27 Gy, 9 Gy/fraction (red line) and peripheral dose 9 Gy, 3Gy/fraction (orange line).

The patient was evaluated to receive chemotherapy combined with external beam radiation therapy (EBRT). Due to the large size and the radioresistant nature of the tumor, lattice radiation technique (LRT) was considered as the best option of treatment as follows:

LRT: 3 Gy (peripheral dose) + 9 Gy in vertices: 3 fractions. The vertices size was between 1.5 - 2 cm and the number of structures were 12. Treatment planning is shown in Figure 2.

After LRT, the patient received:

1.8 Gy: 5 fractions.

2 Gy (peripheral dose) + 5 Gy as IB: 5 fractions

1.8 Gy: 5 fractions

2 Gy (peripheral dose) + 5 Gy (strips): 5 fractions.

**Figure 3:**
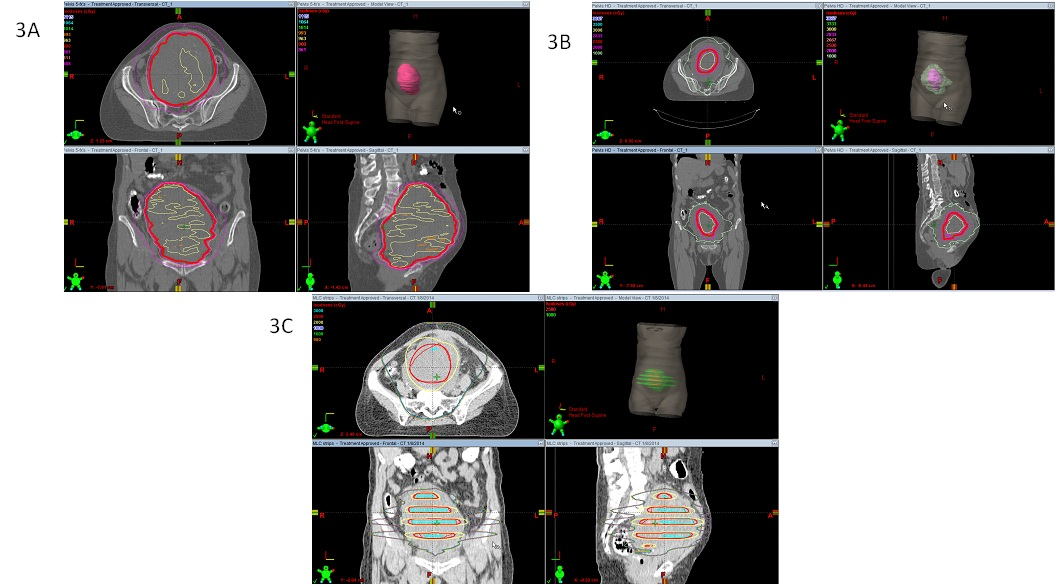
Sequence of the three subsequent treatment plans Treatment plans after LRT. 3A)  1.8 Gy x 5 fractions: Total dose: 7.2 Gy. 3B) Integrated boost (IB): 2 Gy (peripheral dose-green line) + 5 Gy (IB-pink line) x 5 fractions. Total dose: 10 Gy (25 Gy IB). 3C) 2 Gy (peripheral dose)+ 5Gy (strips) x 5 fractions. Total dose: 10 Gy and 25 Gy (strips).

The scheme of treatment, as described above in Table 1, was continuously changing with the intent of delivering doses as high as possible inside the bulky tumor without altering the peripheral dose. Figure 3 shows the sequence of the three subsequent treatment plans after LRT.

**Table 1:** Scheme of treatment: procedures and doses LTR (lattice radiation therapy).  IB (integrated boost)

Procedures	Fractions	Peripheral Dose	High Dose
LTR (Fig 1)	3	9 Gy	27 Gy
1.8 Gy x 5fx (Fig 3a)	5	9 Gy	9 Gy
IB (Fig 3b)	5	10 Gy	25 Gy
1.8 Gy x 5fx (Fig 3a)	5	9 Gy	9 Gy
Strips (Fig 3c)	5	10 Gy	25 Gy
Total Dose	23	47 Gy	95 Gy

In the treatment planning, the gross tumor volume (GTV) was contoured using a planning CT diagnostic image. Planning target volume (PTV) margins were set at 5 mm. Treatment was delivered with image-guided radiation therapy (IGRT) using cone beam CT (CBCT) in every fraction using the Varian Trilogy™ linear accelerator (Varian Corp, Palo Alto, CA). A volumetric modulated arc therapy (VMAT) technique was used. Fractions of treatment were given in consecutive days. In total, 23 fractions were delivered in 43 elapsed days. She completed the treatment in January 2014 without any significant toxicity.

**Figure 4:**
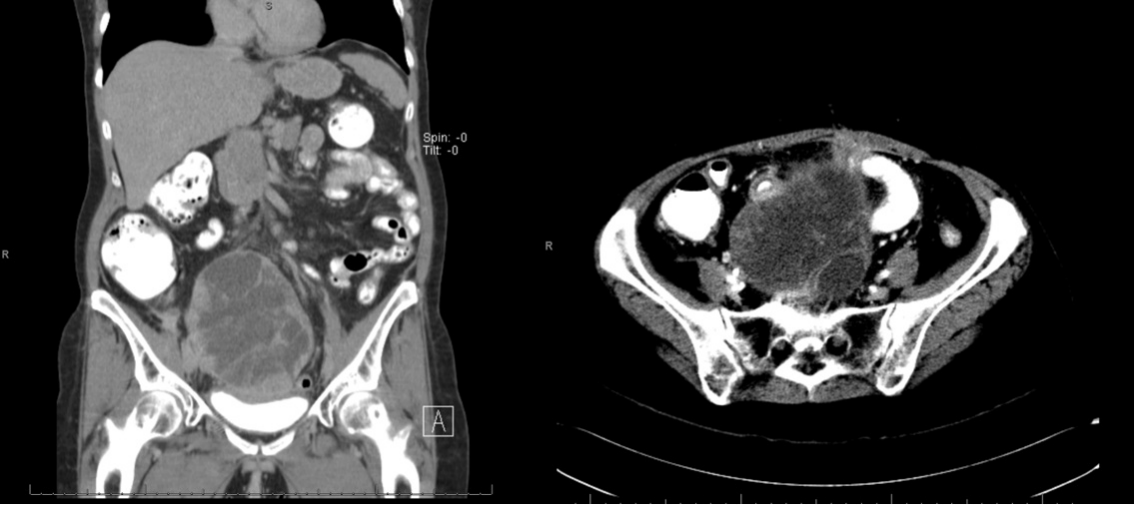
Abdominal CT scan at first follow-up Abdominal CT shows interval decrease in size of the large heterogeneous pelvic mass 4 months after radiation therapy. (February 2014)

The first follow-up visit one month after treatment in February 2014 showed an interval decrease in the size of the large heterogeneous pelvic mass and the tumor implant along the right psoas muscle in CT of the abdomen. This decrease in the size of the pelvic mass is shown in Figure 4.

**Figure 5:**
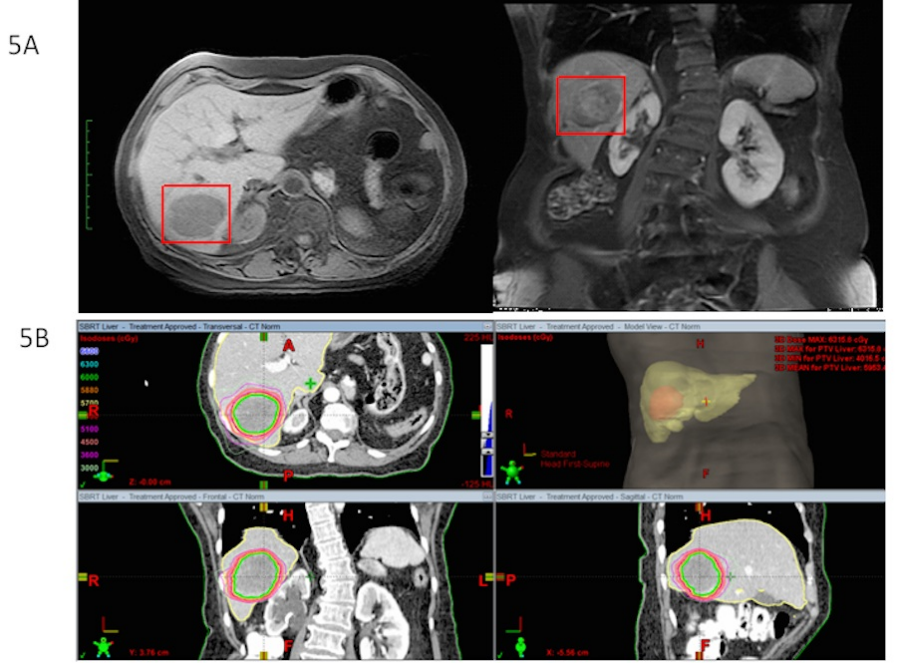
Liver MRI and SBRT Treatment Planning 5A) Large lesion in the right lobe of the liver (segment 7/6). 5B) Treatment planning SBRT for liver metastasis. Dose: 20 Gy x 3 fractions. Total dose: 60 Gy.

In September 2014, MRI of the abdomen showed a large lesion in the right lobe of the liver (segment 7/6) and interval decrease of the pelvic mass. The patient had a liver lesion biopsy, which revealed metastatic adenocarcinoma consistent with adenocarcinoma of Müllerian origin. She was treated with SBRT to the liver metastases, receiving a total dose of 60 Gy in 3 fractions (20 Gy per fraction). Figure 5 shows the diagnostic MRI of the liver and treatment plan for SBRT liver metastasis with different isodose lines.

**Figure 6:**
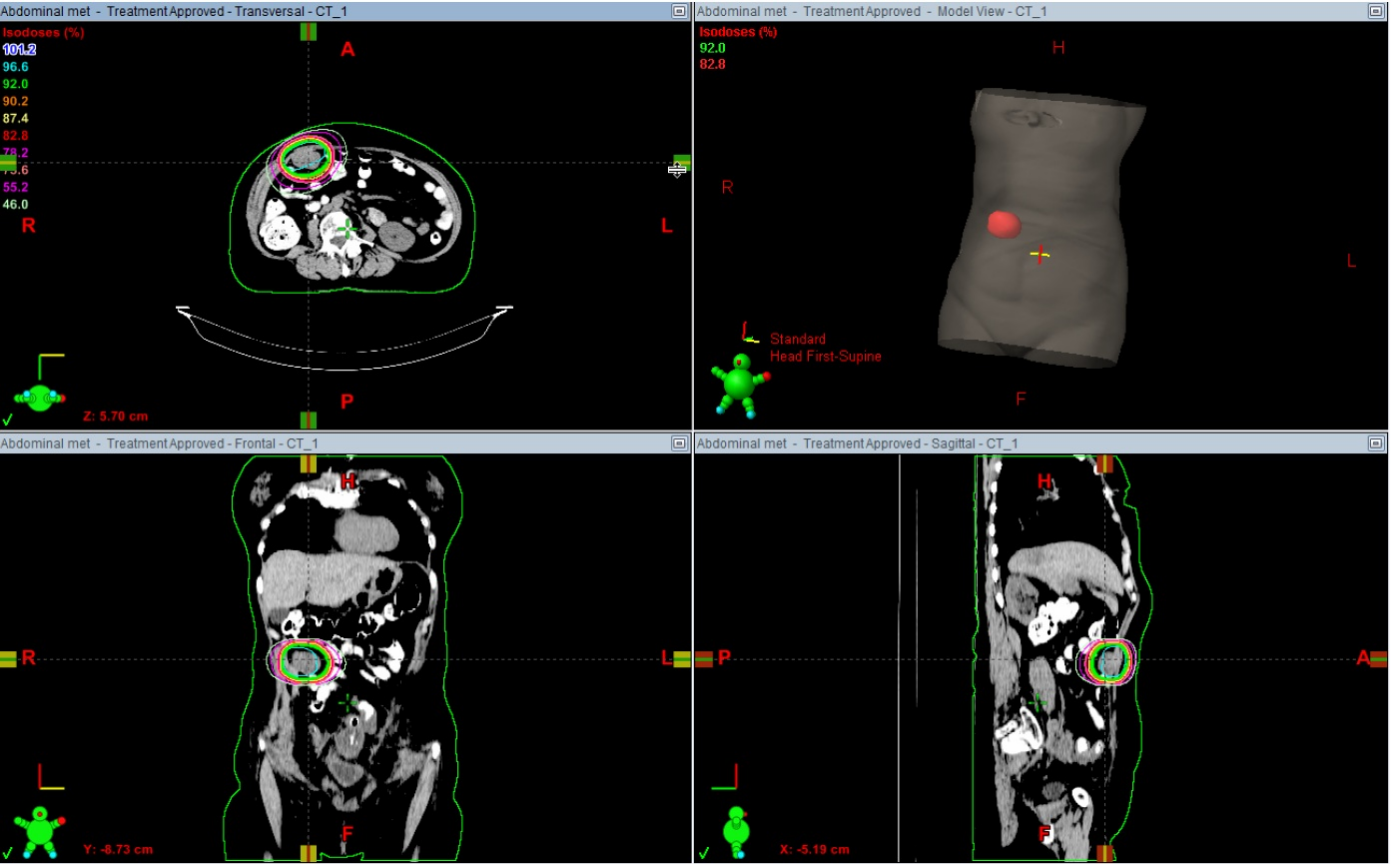
Recurrent abdominal mass treatment planning Treatment planning for recurrent abdominal mass. Dose: 2.5 Gy x 15 fractions. Total dose: 37.50 Gy.

Three months later, in December 2014, new studies demonstrated a new abdominal wall mass with stable disease in the previously treated areas. In the physical examination, she had a palpable firm abdominal wall mass with no pain. The patient underwent retreatment with radiation therapy to the metastatic lesion in the abdominal wall. She completed treatment to the abdominal wall metastasis using a conformal partial arc. Total dose delivered was 37.50 Gy using 2.5 Gy per fraction in 15 fractions. She tolerated the treatment well with no morbidity. Figure 6 shows the treatment plan.

**Figure 7:**
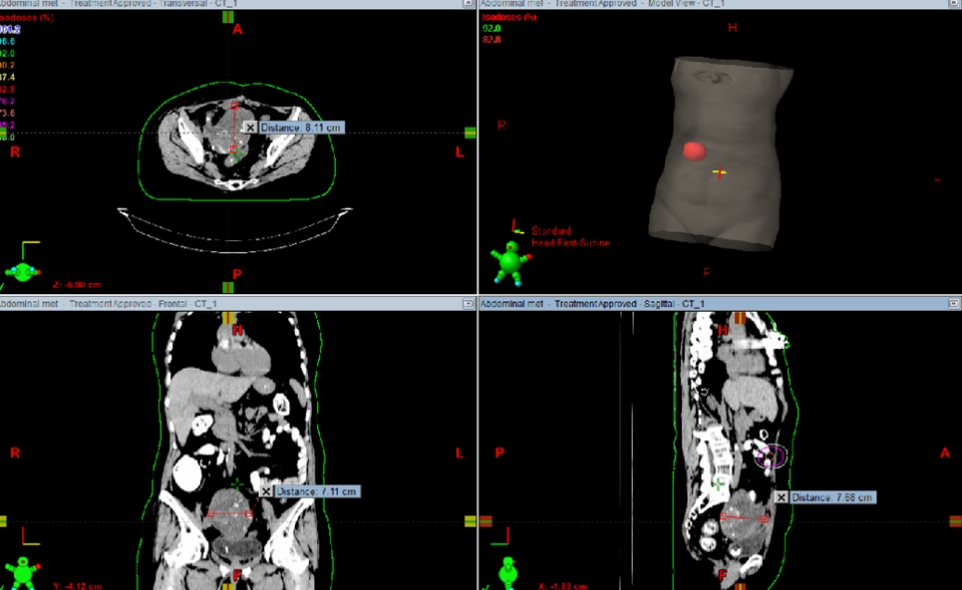
Abdominal CT scan follow-up A decrease in size of the abdominal mass treated previously. The volume of the lesion has decreased from 1495 cc to 442.8 cc (over 70%).

Her last CT scan of the abdomen/pelvis in June 2015 demonstrated a decrease in the size of the liver lesions, a decrease in the size of the soft tissue mass identified within the abdominal cavity right of midline, and the large calcified pelvic mass. The volume of the lesion has decreased from 1.495 cc to 442.8 cc, over 70%, as shown in Figure 7.

According to the last follow-up in August 2015, three years after treatment completion, the patient had stable disease with a Karnofsky performance status (KPS) of 90 %. The patient died in October 2015 due to distant disease progression with local control in the abdominal lesions previously treated, a good performance status (KPS 90%), and quality of life. Overall survival was 50 months from diagnosis (four years and two months).

## Discussion

Wu, et al. [7] described the physics basis of this technique, which improves the response of bulky tumors triggering the bystander effect in the normal cells adjacent to a tumor, where a very high dose of radiation is given to small areas inside the tumor. Amendola, et al. presented the clinical results in a patient treated for a bulky cervical cancer with LRT showing complete local response [8]. In the present case presentation, we describe the use of LRT applied with modern radiotherapy techniques and combined with chemotherapy as a new approach to improve treatment results in bulky tumors. The additional use of LRT technique up front delivering inhomogeneous dose distribution combined with systemic chemotherapy and standard fractionated radiation therapy demonstrated the value of this technique in the management of advanced bulky tumors. It is proven to be highly effective regarding local control with low-grade toxicity, as shown in the patient’s clinical and imaging follow-up. This is supported also by the encouraging results obtained in other patients treated with this technique in our clinic [8]. Additional research is needed in order to generalize our results.

## Conclusions

Lattice radiation therapy is a novel, safe, and effective technique that can be used for the treatment of voluminous tumors (size > 6 cm), allowing for the delivery of a very high dose inside the tumors. This means a higher local control without adding any extra toxicity in the peripheral normal tissue regions. LRT combined with chemotherapy for the treatment of the large recurrent ovarian mass in this patient was very well tolerated. It proved to be an optimal therapeutic option demonstrated with excellent clinical and image-based follow-up results for more than four years.

## References

[1] Berton-Rigaud D, Devouassoux-Shisheboran M, Ledermann JA, Leitao MM, Powell MA, Poveda A, Beale P, Glasspool RM, Creutzberg CL, Harter P, Kim JW, Reed NS, Ray-Coquard I (2014). Gynecologic Cancer InterGroup (GCIG) consensus review for uterine and ovarian carcinosarcoma. Int J Gynecol Cancer.

[2] Amant F, Cadron I, Fuso L, Berteloot P, de Jonge E, Jacomen G, Van Robaeys J, Neven P, Moerman P, Vergote I (2005). Endometrial carcinosarcomas have a different prognosis and pattern of spread compared to high-risk epithelial endometrial cancer. Gynecol Oncol.

[3] Garg G, Shah JP, Kumar S, Bryant CS, Munkarah A, Morris RT (2010). Ovarian and uterine carcinosarcomas: a comparative analysis of prognostic variables and survival outcomes. Int J Gynecol Cancer.

[4] Pacaut C, Bourmaud A, Rivoirard R, Moriceau G, Guy JB, Collard O, Bosacki C, Jacquin JP, Levy A, Chauleur C, Magné N, Merrouche Y (2015). Uterine and ovary carcinosarcomas: outcome, prognosis factors, and adjuvant therapy. Am J Clin Oncol.

[5] Rauh-Hain JA, Growdon WB, Rodriguez N, Goodman AK, Boruta DM 2nd, Schorge JO, Horowitz NS, del Carmen MG (2011). Carcinosarcoma of the ovary: a case-control study. Gynecol Oncol.

[6] Doss LL, Llorens AS, Henriquez EM (1984). Carcinosarcoma of the uterus: a 40-year experience from the state of Missouri. Gynecol Oncol.

[7] Wu X, Ahmed MM, Wright J, Gupta S, Pollack A (2010). On modern technical approaches of three-dimensional high dose Lattice radiotherapy (LRT). Cureus.

[8] Amendola B E, Perez N, Amendola M, Wu X, Ahmed MM, Iglesias AJ, Estape R, Lambrou N, Bortoletto P (2010). Lattice radiotherapy with rapidArc for treatment of gynecological tumors: dosimetric and early clinical evaluations. http://www.cureus.com/articles/17-lattice-radiotherapy-with-rapidarc-for-treatment-of-gynecological-tumors--dosimetric-and-early-clinical-evaluations.

